# Case report: A new *de novo* 6q21q22.1 interstitial deletion case in a girl with cerebellar vermis hypoplasia and developmental delay and literature review

**DOI:** 10.3389/fgene.2023.1315291

**Published:** 2024-02-06

**Authors:** Chiara Minotti, Ludovico Graziani, Ester Sallicandro, Maria Cristina Digilio, Roberto Falasca, Viola Alesi, Giuseppe Novelli, Maria Lisa Dentici, Sara Loddo, Antonio Novelli

**Affiliations:** ^1^ Medical Genetics Unit, Translational Pediatrics and Clinical Genetics Research Area, Bambino Gesù Children Hospital, Istituto di Ricovero e Cura a Carattere Scientifico, Rome, Italy; ^2^ Medical Genetics Section, Depepartment of Biomedicine and Prevention, Tor Vergata University of Rome, Rome, Italy; ^3^ Translational Cytogenomics Research Unit, Bambino Gesù Children’s Hospital, IRCCS, Rome, Italy; ^4^ Medical Genetics Lab, Tor Vergata Hospital, Rome, Italy

**Keywords:** 6q21q22.1, interstitial deletion, cerebellar vermis hypoplasia, chromosomal microarray analysis, developmental delay

## Abstract

Interstitial deletions involving 6q chromosomal region are rare. Less than 30 patients have been described to date, and fewer have been characterized by high-resolution techniques, such as chromosomal microarray. Deletions involving 6q21q22.1 region are associated with an extremely wide and heterogeneous clinical spectrum, thus genotype–phenotype correlation based on the size of the rearranged region and on the involved genes is complex, even among individuals with overlapping deletions. Here we describe the phenotypic and molecular characterization of a new 6q interstitial deletion in a girl with developmental delay, intellectual disability, cerebellar vermis hypoplasia, facial peculiar characteristics, ataxia and ocular abnormalities. Microarray analysis of the proposita revealed a 7.9 Mb interstitial *de novo* deletion at 6q21q22.1 chromosomal region, which spanned from nucleotides 108,337,770 to 116,279,453 (GRCh38/hg38). The present case, alongside with a systematic review of the literature, provides further evidence that could aid to the definition of the Smallest Region of Overlap and of the genomic traits that are associated with particular phenotypes, focusing on neurological findings and especially on cerebellar anomalies.

## Introduction

Deletions of the long arm of chromosome 6 are rare, and less than 30 patients with interstitial deletions involving the 6q21q22.1 region have been described to date (Schwartz et al., 1984; Young et al., 1985; Pandya et al., 1995; Correa-Cerro et al., 1996; Evers et al., 1996; Hopkin et al., 1997; Tsukahara et al., 1997; Duran-Gonzalez et al., 2007; Zherebtsov et al., 2007; Rosenfeld et al., 2012; Toschi et al., 2012; Hudson et al., 2014; Szafranski et al., 2015; Tassano et al., 2015; Milani et al., 2016; Shukla et al., 2016; Donahue and Rohena, 2017; Machida et al., 2022).

Hopkin et al. (1997) first classified deletions of the long arm of chromosome 6 into three groups, based on conventional cytogenetics, with different and recurrent phenotypes: group A, or proximal deletions (6q11q16), group B, or medial deletions (6q15q25), and group C, or terminal deletions (6q25qter) (Hopkin et al., 1997).

Intellectual disability, developmental delay, hypotonia and postnatal growth retardation appear to be common and non-specific features among patients with 6q deletions (Tassano et al., 2015; Hopkin et al., 1997). Medial deletions (6q15q25) are associated with additional recurrent clinical features including intrauterine growth restriction (IUGR), abnormal respiration, hypertelorism, ear anomalies and upper limb malformation (Hopkin et al., 1997; Donahue and Rohena, 2017). Nonetheless, no univocal genotype-phenotype correlation has been determined so far, even comparing overlapping 6q deletions.

Standard cytogenetic techniques were performed in the first reports (Schwartz et al., 1984; Young et al., 1985; Pandya et al., 1995; Correa-Cerro et al., 1996; Evers et al., 1996; Tsukahara et al., 1997; Duran-Gonzalez et al., 2007; Hopkin et al., 1997). More recent studies are based on higher resolution techniques, such as chromosomal microarray analysis (CMA) (Zherebtsov et al., 2007; Rosenfeld et al., 2012; Toschi et al., 2012; Hudson et al., 2014; Szafranski et al., 2015; Tassano et al., 2015; Milani et al., 2016; Shukla et al., 2016; Donahue and Rohena, 2017; Machida et al., 2022), which has allowed a better characterization of the deleted region and of the genes involved, as new and different clinical features emerged in patients described over the years.

Here, we report a girl with a 6q21q22.1 *de novo* deletion, detected by CMA, and we focus on her neurological findings detected by brain MRI, such as cerebellar vermis hypoplasia (CBVH). We also provide a review of the literature of the reported cases with overlapping rearrangements.

## Case report

The patient is a 2-year-old girl, addressed to our Genetics Unit due to developmental delay. She is the only child of healthy non-consanguineous parents with unremarkable family history.

She was born at 41 weeks of gestational age by cesarean section due to fetal distress, after an uneventful pregnancy. Standard chromosome analysis performed prenatally was normal (46, XX). Birth weight, length and occipitofrontal circumference (OFC) were 3.000 gr (−0.92 SDS), 49 cm (−0.13 SDS), and 35 cm (0.85 SDS) respectively. The Apgar score was 9 at 1 min and 10 at 5 min.

Gross motor development delay was reported: she was able to sit with no support at 8–9 months and she could walk independently at 20 months. She presented a speech delay, as she could pronounce her first words at about 2 years of age and was unable to complete intelligible sentences at the time of examination.

At physical examination (2 years old), her weight was 16 kg (1.23 SDS), her height was 99 cm (1.88 SDS) and her OFC was 49.3 cm (0.82 SDS). Phenotypic features included hypertelorism, downslanted palpebral fissures, epicanthal folds, prominent nasal bridge, low-set small ears with thick helix, bilateral pes valgus, and mild generalized hypotonia.

Ophthalmologic evaluation documented oculomotor apraxia and right convergent strabismus. Neurological evaluation demonstrated general clumsiness and ataxia manifested by balance deficit and a wide-based gait, which her parents referred to be congenital.

**FIGURE 1:**
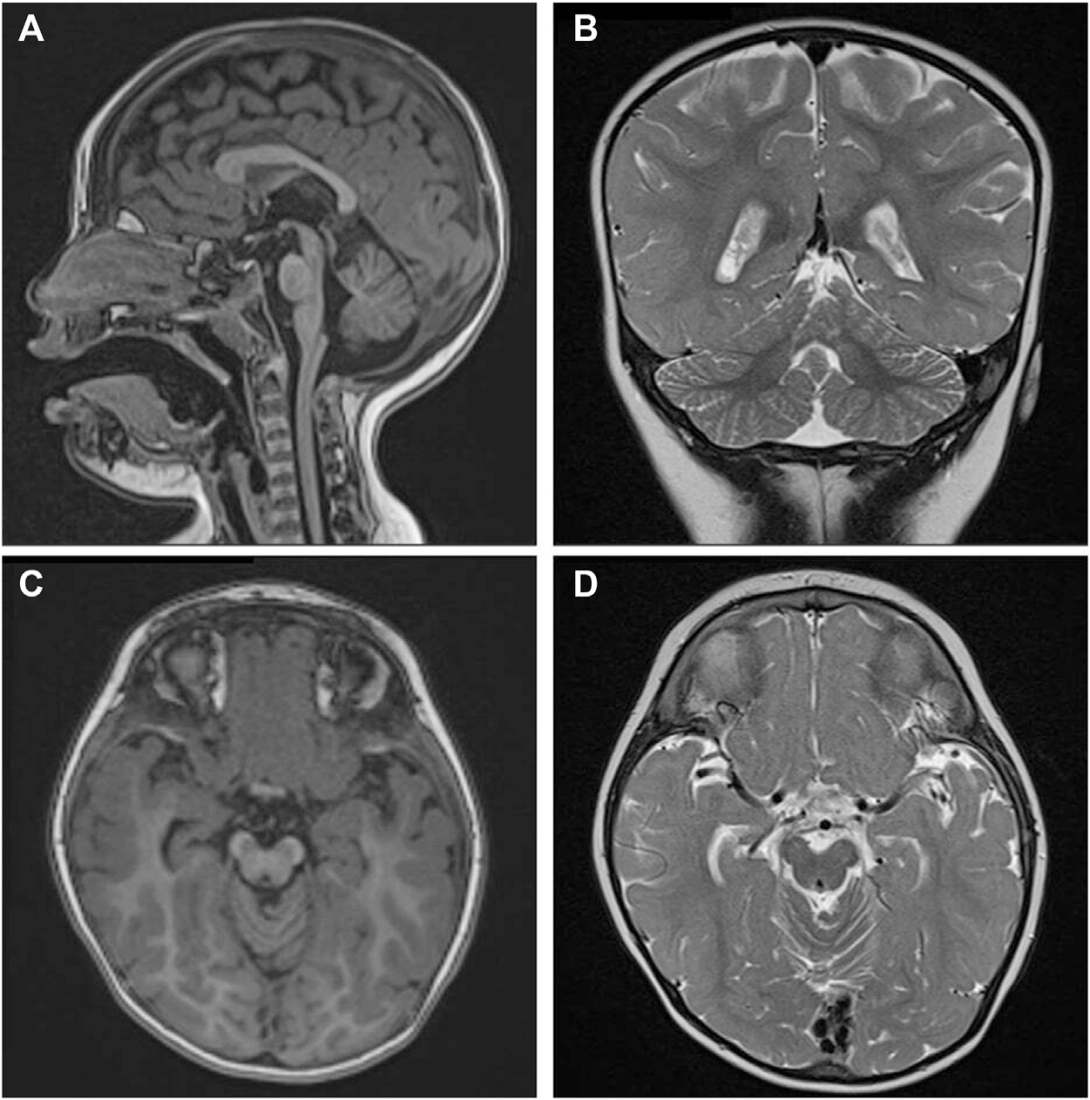
Brain Magnetic Resonance imaging (MRI) analyses of the patient. **(A)** Mid sagittal T1-weighted image and **(B)** Coronal T2-weighted image demonstrating cerebellar vermis hypoplasia **(B)**. **(C)** Axial T1-weighted and **(D)** T2-weighted images showing mild asymmetry of cerebral peduncles.

Brain MRI (Magnetic Resonance Imaging) at 2 years old revealed CBVH, cerebellar volume reduction and a minimal asymmetry of cerebral peduncles (Figure 1).

According to Griffiths Mental Development Scales (GMDS) (Griffiths, 1970), our patient presented with a General Quotient (GQ) score of 79 and a developmental age of 18 months *versus* a chronological age of 23 months. Her major deficits consisted in poor oculo-manual coordination and attention defects.

Informed consent was obtained from the parents of the proposita, and CMA was performed. The analysis revealed a 7.9 Mb interstitial deletion at 6q21q22.1 chromosomal region, which spanned from nucleotides 108,337,770 to 116,279,453 (GRCh38). FISH (Fluorescence *In Situ* Hybridization) on metaphase chromosome preparation from cultured lymphocytes of patient and her parents confirmed a highly likely *de novo* deletion.

## Materials and methods

All data were obtained in agreement with Bambino Gesù Children Hospital ethical standards. CMA was performed using Infinium CytoSNP-850K BeadChip (Illumina, San Diego, CA), according to the manufacturer’s protocol. Array scanning data were generated on the Illumina NextSeq 550 system and the results were analyzed by the BluefuseMulti 4.4 software. Confirmation and segregation tests were performed by FISH on metaphase chromosome preparations of the patient and her parents, using different locus-specific BAC probes [RP11-469I15 (6q21) e RP11-179F7 (6q22.1)]. BACs clones were selected from a genomic library (32 K library; BACPAC Resources, Oakland, CA).

We searched the DECIPHER database and scientific reports on PubMed to identify individuals with overlapping chromosomal imbalances characterized through postnatal CMA, specifically involving the 6q21q22.1 chromosomal region.

## Discussion

Interstitial deletions of the 6q region are related with an extremely wide phenotypic spectrum. Intellectual disability, developmental delay, facial peculiar characteristics, hypotonia and postnatal growth retardation are frequent but non-specific features of the condition, and thus present in most chromosome imbalances (Zherebtsov et al., 2007; Hopkin et al., 1997).

We report a new interstitial 6q deletion in a girl with developmental delay, hypotonia, ataxia, facial peculiar characteristics, CBVH and skeletal, ophthalmological, and neurological anomalies.

Although numerous 6q21q22.1 deletion cases have been described, only the most recent ones have been characterized with molecular cytogenetics resolution. In most patients, CMA techniques have demonstrated that chromosomal rearrangements show non-recurrent breakpoints and only partial overlap (Rosenfeld et al., 2012; Szafranski et al., 2015; Milani et al., 2016). The following mechanisms could be hypothesized for the non-recurrent breakpoints observed: Non-Allelic Homologous Recombination (NAHR), Non-Homologous End Joining (NHEJ) or Fork Stalling and Template Switching (FoSTeS) (Gu et al., 2008; Stankiewicz and Lupski, 2002; Zhang et al., 2009). Notwithstanding these limitations, previous authors suggested some genotype-phenotype correlations for 6q interstitial deletion.

Rosenfeld et al. (2012) described a cohort of 12 individuals with variable deletions within the 6q15q22.33 region and compared their clinical features. Heterogeneous clinical expression was reported, even among individuals with overlapping deletions. Some facial peculiar characteristics were variably shared among different cases, with hypertelorism, microcephaly and broad/flat nasal bridge being the most common (Rosenfeld et al., 2012).

Toschi et al. (2012) and then Hudson et al. (2014) described the association between 6q21q22.3 microdeletions and Acrocardiofacial syndrome (ACFS) (Toschi et al., 2012; Hudson et al., 2014). Developmental anomalies of muscular tissue (as in Poland syndrome) have also been reported (Tassano et al., 2015). Milani et al. (2016) hypothesized a critical region for ACFS or at least congenital heart disease (CHD), spanning from nucleotides 107,754,749 to 110,769,883 (GRCh38). They also pointed out a possible role of three genes: *SNX3* (MIM*605930), *SESN1* (MIM*606103) and *FOXO3* (MIM*602681) (Milani et al., 2016). Shukla et al. (2016) reported on a patient who shared similar deletion breakpoints with those described by Toschi et al. and Hudson et al., but lacked any feature suggestive of ACFS (Shukla et al., 2016). We as well describe a patient who does not display any phenotypic feature suggestive of ACFS. This may underline the extreme phenotypic variability in this microdeletion syndrome and also could raise doubts on whether ACFS belongs to the group of microdeletion syndromes (Shukla et al., 2016).

Some authors speculated that interstitial deletion in the 6q21q22.1 region could be a risk factor for structural neurological anomalies, mainly involving the corpus callosum (CC) and the lateral and the third ventricles (Rosenfeld et al., 2012; Toschi et al., 2012; Shukla et al., 2016; Donahue and Rohena, 2017). Furthermore, Szafranski et al. (2015) suggested that the 6q22 region contains important contributors to the onset of childhood epilepsy (Szafranski et al., 2015).

**FIGURE 2:**
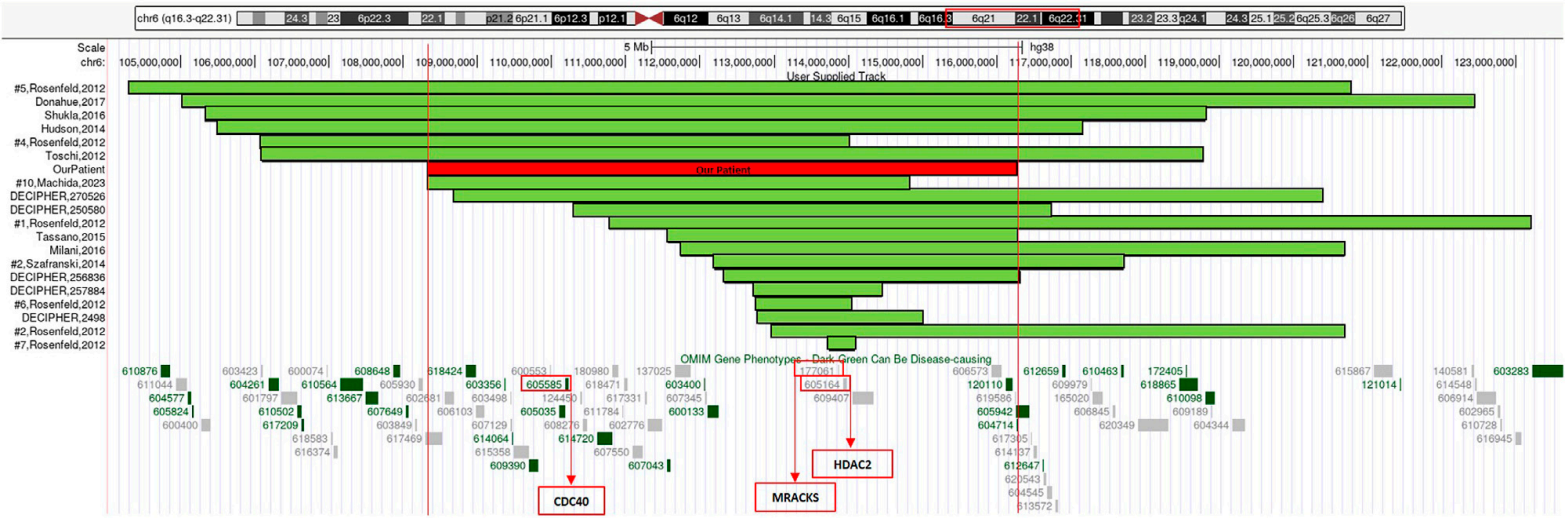
6q21q22.1 deletions aligned according to the proximal breakpoint, reference articles are reported on the left, our patient is outlined in red. At the bottom of the figure is an overview of the region 6q21q22.1 and its OMIM gene phenotypes content according to the UCSC Genome Browser [GRCh38/hg38 assembly]. In red boxes we highlighted three genes considered to be relevant in neurological developmental disorders and in cerebellar development.

Our patient’s rearrangement contains 90 genes, 13 of which are classified, to current knowledge, as OMIM (Online Mendelian Inheritance in Man) disease-associated (Figure 2): *ARMC2* (MIM*618424) armadillo repeat-containing protein 2; *CCN6* (MIM*603400) cellular communication network factor 6; *CD164* (MIM*603356) CD164 antigen; *CDC40* (MIM*605585) cell division cycle 40; *CDK19* (MIM*614720) cyclin-dependent kinase 19; *COL10A1* (MIM*120110) collagen, type X, alpha-1; *DSE* (MIM*605942) dermatan sulfate epimerase; *FIG4* (MIM*609390) FIG4 phosphoinositide 5-phosphatase; *LAMA4* (MIM*600133) laminin, alpha-4; *TRAF3IP2* (MIM*607043) TRAF3-interacting protein 2; *TSPYL1* (MIM*604714) TSPY-like 1; *WASF1* (MIM*605035) WASP protein family, member 1; *ZBTB24* (MIM*614064) zinc-finger and BTB domain-containing protein 24.

We considered the shared 6q21q22.1 chromosomal region to compare our patient’s phenotype with the other cases characterized by CMA in scientific literature and within genomic databases (e.g., DECIPHER) (Table 1) (Figure 2).

**TABLE 1:** Comparison of the reported case with individuals with overlapping chromosomal deletions involving the 6q21q22.1 chromosomal region, characterized through postnatal CMA.

Patient’s data		Our Patient	Milani et al. (2016)	Tassano et al. (2015)	#1 Rosenfeld et al. (2012)	#2 Rosenfeld et al. (2012)	#4 Rosenfeld et al. (2012)	#5 Rosenfeld et al. (2012)	#6 Rosenfeld et al. (2012)	#7 Rosenfeld et al. (2012)	DECIPHER N. 2498
Deletion Interval (UCSC GRCh38)		chr6:108337770-116279453	chr6:111736995-120684991	chr6:111563437-116273478	chr6:110769883-123191957	chr6:112980094-120673658	chr6:106078319-114022551	chr6:104297784-120745892	chr6:112762166-114022551	chr6:113742769-114086116	chr6:112785705-115004771
Dimension (bp)		7.9 Mbp	8.9 Mbp	4.7 Mbp	1.2 Mbp	7.7 Mbp	7.9 Mbp	16.5 Mbp	1.3 Mbp	343.3 kbp	2.2 Mbp
Inheritance		*De novo*	Unknown	Mother normal/father NA	Unknown	*De novo*	*De novo*	*De novo*	Unknown	*De novo*	Unknown
Age (at the time of examination)		2 y and 8 m	13 y	12 y	6.5 y	33 y	12 y	10.5 m	12 y	5 y	NA
Gender		Female	Male	Male	Male	Male	Male	Female	Male	Male	NA
OFC	at birth	0.85 SDS	−1.25 SDS	NA	NA	NA	NA	NA	NA	NA	NA
	present age	0.82 SDS	<−2 SDS	−2 SDS/−1.25 SDS	−4.3 SDS	0 SDS	−2.4 SDS	−5.4 SDS	−1.4 SDS	−2.3 SDS	−7.2 SDS
Weight	at birth	−0.65 SDS	−1.25 SDS/−0.65 SDS	1.25 SDS	NA	−0.65 SDS	−1.25 SDS/−0.65 SDS	−2.5 SDS	0 SDS/0.65 SDS	0.65 SDS/0 SDS	NA
	present age	1.25 SDS/2 SDS	−2 SDS	0 SDS/0.65 SDS	NA	1.25 SDS	0.65 SDS/1.25 SDS	−2.8 SDS	0.15 SDS	0 SDS	0.4 SDS
Height	at birth	−0.65 SDS/0 SDS	0 SDS/0.65 SDS	NA	NA	NA	NA	NA	NA	NA	NA
	present age	1.25 SDS/2 SDS	−2 SDS	1.25 SDS/2 SDS	NA	0.65 SDS	0 SDS/0.65 SDS	−1.25 SDS/−0.65 SDS	0 SDS	−0.65 SDS/0 SDS	−1.2 SDS
Cerebellar malformations	CBVH	+	-	+	-	Mild	-	+	-	+	-
	Other	Cerebellar peduncles asimmetry, minimal reduction in cerebellar volume	-	-	-	-	Small cerebellum	-	-		-
Brain malformations		Parietal arachnoid cysts, T2/FLAIR hyperintensity of the periventricular white matter	Mild asymmetry of the lateral ventricle and moderate thickening of the frontal cortex	-	-	-	Partial CC hypoplasia, generally underdeveloped brain	Underdeveloped genu of CC	-	Periventricular leukomalacia, thick CC	Thin corpus callosum, reduction in volume in the left hemi-cranium with cystic cerebromalacia
ID/DD		Mild	Moderate	Mild	+	Mild-moderate	+	+	Mild	+	+
Behavioural Disorders		-	Irritability, low frustration tolerance, provocative attitude	-	Severe ADHD	-	-	-	-	ADHD	-
Epilepsy (age of onset)		-	History of febrile convulsions, absence-like episodes, absences with automatisms, and myoclonic seizures (NA)	-	- (One complex partial seizure)	- (One FS at 8 months)	-	Infantile spasms, GTCS, FS, myoclonic jerks (NA)	-	- (One seizure-like episode)	+ (NA)
Hypotonia		+	+	-	-	-	-	+	-	-	+
Ataxia/Gait anomalies		+	+	-	+	+	-	-	-	-	-
Other Neurological disorders	Clumsiness	+	+	Mild	-	-	-	-	-	-	-
	Other	-	Lower limb distal hypertonia, hyperkinetic and stereotyped movements	-	-	Adult-onset reticular myoclonus, dysmetria, fine tremor of upper extremities	Holds hands clenched	Unusual finger positioning, poor head control	-	Poor articulation	Right hemiplegia with brisk reflexes and spasticity
Ocular Findings	Oculomotor apraxia	+	-	-	-	-	-	-	+	-	-
	Strabismus	+	+	+	-	-	+	+	+	+	-
	Myopia	-	-	+	-	+	-	-	-	-	-
	Nystagmus	-	-	-	-	-	-	+	-	-	-
	Other	-	-	-	-	-	Bilateral colobomas	Nystagmus, Poor visual processing, abnormal pupil dilation	-	Unilateral Duane anomaly	Moderate cerebral visual impairment
Dysmorphic features		Hypertelorism, downslanting palpebral fissures, marked bilateral epicanthal folds, prominent nasal bridge, low-set small ears with thick helix	Trigonocephaly, low forehead, elongated and dysmorphic ears, arched eyebrows, high nasal root, tubular nose, epicanthus, downslanting palpebral fissures, downward columella, short philtrum	Hypertelorism, wide and flat nose	Bitemporal hollowing, bilateral ear pits, highly arched and disrupted eyebrows	Two hair whorls, oval facies, small forehead with low frontal hairline, broad nasal bridge, long columella, mild retrognathia, high and narrow palate	Small anterior fontanelle, mild brachycephaly, bitemporal narrowing, small, posteriorly rotated, cupped ears with unraveled helices, epicanthal folds, hypoplastic supraorbital ridges, hypertelorism, downslanting palpebral fissures, wide nasal bridge, small jaw, cupid bow upper lip, long philtrum	Brachycephaly, unraveled helices, long eyelashes, downslanting palpebral fissures, prominent nasal tip, thick alae nasi, downturned mouth corners, thick upper maxillary frenulum, mildly short philtrum	Hypertelorism, wide nasal bridge, narrow nasal tip, long nose	Mildly arched eyebrows, pointed chin with dimples	Hypertelorism, prominent simple ears, highly arched eyebrow, underdeveloped supraorbital ridges
Musculoskeletal features		-	-	Pectus excavatum, chest asimmetry, absence of the pectoralis major and minor muscles asymmetry, thoracic scoliosis and vertebral rotation	-	Pectus excavatum, scoliosis, hyperextensible joints	Small chest wall musculature	Minimal pectus excavatum	Pectus carinatum, hyperextensible joints	-	-
Limbs features		Bilateral pes valgus	-	-	Extra creases on fingers and toes	Broad feet, hypoplastic 5th toenails	Camptodactyly at the proximal finger interphalangeal joints, absence of the 4th distal finger flexion crease, narrow and hyperconvex nails	Middle finger camptodactyly, hyperconvex nails, narrow feet with jumbled toes	Long and slender fingers and toes	-	Required reconstruction of right hip probably secondary to hemiplegia
Other features not previously specified		-	-	-	-	Small penis, pronounced gynecomastia	DORV, dysplastic pulmonary valve, pulmonary atresia, large VSD and ASD, unilateral duplicated collecting system, hydronephrosis	PDA, recurrent ear infections, delayed gastric emptying, GERD, nasolacrimal duct stenosis, decreased muscle mass	Hernia, absent testicle, 4 café au lait macules	Undescended testes, multiple flame nevi on face and back	Constipation

DECIPHER N. 250580	DECIPHER N. 256836	DECIPHER N. 257884	DECIPHER N. 270526	Toschi et al. (2012)	Hudson et al. (2014)	Donahue et al. (2017)	Shukla et al. (2016)	#2 Szafranski et al. (2015)	#10 Machida et al. (2022)	Total: 20	%
chr6:110285784-116729774	chr6:112328359-116326501	chr6: 112723203-114450824	chr6:108690284-120404690	chr6:106095668-118788209	chr6:105482175-117161934	chr6:105017513-122451895	chr6:105323645-118809642	chr6:112190549-117716432	chr6:108349530-114822633	-	-
6.44 Mbp	4.0 Mbp	1.7 Mbp	11.71 Mbp	12.5 Mbp	11.5 Mbp	17.3 Mbp	13.4 Mbp	5.5 Mbp	6.5 Mbp	-	-
*De novo*	Inherited from parent (with similar phenotype)	*De novo*	*De novo*	*De novo*	*De novo*	*De novo*	*De novo*	*De novo*	NA	-	-
6 y	1 y	5 y	2 y	Newborn	2 m	18 m	7 y	4 y	3 y and 10 m	-	-
Female	Female	Female	Female	Male	Female	Male	Male	Male	Female	11M 8F 1NA	55%M 40%F 5%NA
NA	NA	NA	NA	−2 SDS/−0.65 SDS	−1.25 SDS/−0.65 SDS	−2 SDS/−1.25 SDS	−0.65 SDS/0 SDS	NA	NA		
Microcephaly	NA	−2 SDS	Microcephaly	−2 SDS/−0.65 SDS	−1.25 SDS/−0.65 SDS	>2 SDS	−4 SDS	NA	−1.2 SDS	9/18	50.00%
NA	NA	NA	NA	−0.65 SDS	−2 SDS	<−2 SDS	−0.65 SDS	NA	NA		
NA	NA	−2 SDS	NA	−0.65 SDS	−2 SDS	NA	NA	NA	1.7 SDS	1/13	7.69%
NA	NA	NA	NA	−0.65 SDS	−0.65 SDS	0 SDS	NA	NA	NA		
NA	NA	−1 SDS	NA	−0.65 SDS	−0.65 SDS	NA	0 SDS /0.65 SDS	NA	2.3 SDS	0/14	0.00%
NA	NA	-	+	-	NA	+	-	-	-	7/17	41.18%
		-	-	-		-	-	-	-	-	-
NA	NA	Corpus callosum hypoplasia, cortical dysplasia	NA	Large lateral ventricles, subcortical and periventricular white matter hyperintensities, simplified cortex gyration, slightly thin corpus callosum, cluster of subependymal cysts	NA	Mild dilation of lateral and third ventricles, megacisterna magna and thin, hypoplastic CC	Fronto-temporo-parietal atrophy, mildly dilated ventricles, hypoplastic CC	-	Corpus callosum hypoplasia	-	-
+	+	+	+	+	+	+	+	+	+	20/20	100.00%
-	-	-	Autism spectrum disorder	-	Autism spectrum disorder	-	NA	-	NA	-	-
+ (NA)	-	-	-	-	- (Abnormal EEG with no history of seizures)	Focal epilepsy (18 months)	- (Abnormal EEG with no history of seizures)	-	-	5/20	25.00%
-	-	Moderate	-	+	+	+	-	+	NA	9/19	47.37%
+	-	-	-	-	-	-	-	+	NA	6/19	31.58%
-	-	-	-	-	-	-	-	-	-	3/20	15.00%
Impaired pain sensation	Apraxia	Dysexecutive syndrome	-	-	-	-	-	-	-	-	-
-	-	-	-	-	-	-	-	-	-	2/20	10.00%
-	-	+	-	-	+	-	-	-	-	9/20	45.00%
-	-	-	-	-	-	-	-	-	-	2/20	10.00%
+	-	-	-	-	-	-		-	-	2/20	10.00%
-	-	-	-	-	Hyperopia	-	-	-	-	-	-
Highly arched eyebrow, protruding ear, bulbous nose	Hypertelorism, microtia	Triangular face, retrognathism, low set ears, smooth philtrum	Narrow forehead	High forehead, low-set ears, thin upper helices, prominent inferior crus of the antihelices, underdeveloped antitragus, right preauricular pit fontanel, downslanting palpebral fissures, broad nasal bridge, long philtrum, retro-micrognathia, short neck	Mild brachycephaly, flat forehead, hypertelorism, downslanting palpebral fissures, mild micrognathia, asimmetric ears (deficiency of the crus of the helix and an attached lobule of the left ear; tightly attached lobule and deficiency of the crus of the helix, with prominence of the inferior crus of the right ear)	Brachycephy, large flat anterior fontanel, splaying of the frontal suture, flattened occiput, low-lying anterior hairline with hirsute forehead, downslanting palpebral fissures, tapered ears, anteverted nares, mild micrognathia, short neck	Trigonocephaly, small, low-set and posteriorly rotated ears with deficient helix and prominent anti-helix, downslanting palpebral fissures with epicanthal folds and telecanthus, high arched, flared eyebrows, broad nasal bridge, large widely spaced central incisors, prominent upper frenulum, and short philtrum	Broad and flat nasal bridge, bilateral epicanthal folds, and a mildly high arched palate	Occipital flatness, epicathus, hypertelorism, auricular deformity, wide nasal bridge	-	-
-	-	-	Pectus excavatum	-	Weakness of the neck muscles	External rotation of the shoulders with fixed flexion deformity and elbow webbing	Mild joint laxity	-	-	-	-
Arachnodactyly	-	-	-	Unilateral cleft hand, synostosis of the interphalageal joints of the 5th finger	Bilateral ectrodactyly, absent 4th digit bilaterally	Single-bone forearms with bilateral radii and absent ulnae, right hand with a single metacarpal, a single proximal phalanx, and a bifid distal phalanx, left hand with single metacarpal, single proximal phalanx, and two parallel distal phalanges	Bilateral 5th finger clinodactyly	-	-	-	-
-	Intestinal malrotation	-	-	CHD (PA, VSD, TA)	Early feeding difficulties	Hypoplastic phallus with bilateral cryptorchidism, atrophic buttocks tissue, duodenal atresia, mesocardia without transposition, trivial mitral and tricuspid regurgitation, and PFO with left-to-right shunt, renal hypoplasia, diaphragm eventration	-	-	-	-	-

Abbreviations= ADHD: attention-deficit/hyperactivity disorder; ASD: atrial septal decect; CBVH: cerebellar vermis hypoplasia; CC: corpus callosum; CHD: congenital heart disease; DD: developmental delay; DORV: double outlet right ventricle; EEG: electroencephalogram; F: females; FS: febrile seizure; GERD: gastroesophageal reflux disease; GTCS: generalized tonic–clonic seizure; ID: intellectul disability; M: males; NA: not available; OFC: occipitalfrontal circumference; PA: pulmonary atresia; PDA: patent ductus arteriosus; PFO: patent foramen ovale; TA: truncus arteriosus; VSD: ventricular septal defect.

The only adult patient described to date, patient #2 reported by Rosenfeld et al. (2012), has presented neurological manifestations over time, such as adult-onset reticular myoclonus and fine tremor of upper extremities (Rosenfeld et al., 2012). However, current literature is limited and further evidence is needed to better clarify any possible association with progressive neurological disease.

Epilepsy is often described in association with 6q interstitial deletions (Cutillo et al., 2023). Nonetheless, our patient never presented seizures, nor a pathological EEG was recorded. Reviewing the scientific literature, Szafranski et al. (2015) narrowed down a possible critical region for epilepsy to a 250 kb segment and suggested *NUS1*, *EST AI858607* and *SLC35F1* as candidate genes (Szafranski et al., 2015). This region is not involved in our patient’s rearrangement: this may partially justify the absence of epilepsy in our proposita.

Brain MRI documented the absence of major prosencephalic anomalies in our proposita, and the presence of CBVH with reduced cerebellar volume. These features have been previously reported in other unrelated patients with interstitial 6q deletions who underwent radiological examination (Rosenfeld et al., 2012; Tassano et al., 2015; Donahue and Rohena, 2017). Movement disorders manifested by balance deficit and a wide-based gait in our proposita are well reported in 6q deletion cases with or without CBVH. Inasmuch as to date the development of neuromuscular abnormalities underlying the coordination deficits has not been associated with the pleiotropic effect of a single causative gene: the presence of an oligogenic effect arising within the commonly deleted region has been hypothesized. A defective expression of *GOPC* (Golgi associated PDZ and coiled-coil motif containing; MIM*606845) (cytogenetic location: 6q22.1), which is involved in autophagy and transduction pathways in cerebellar Purkinje cells (Yue et al., 2002), was speculated to play a role in the development of ataxia and abnormal movements, even though it was not included in the deleted region of several patients presenting with CBVH (Rosenfeld et al., 2012). Interestingly, oculomotor apraxia was documented in our patient, and has been rarely reported in previous 6q interstitial deletion case (Rosenfeld et al., 2012).

Hayashi et al. (2017) studied a cohort of 41 patients through Next-Generation Sequencing and CMA techniques to identify candidate genomic aberrations to pontine and cerebellar hypoplasia.

Haploinsufficiency of both *HDAC2* (histone deacetylase 2) (MIM*605164) and *MARCKS* (myristoylated alanine-rich C kinase substrate) (MIM*177061) genes, which are contained in 6q21q22.1 chromosomal deletion, was speculated to be relevant in neurological developmental disorders (Hayashi et al., 2017).

*HDAC2* gene is zinc finger transcription factor (Inouye and Seto, 1994) and plays a role in adult neurogenesis. It is required for full differentiation and survival of adult generated neurons, and it is dispensable during development (Jawerka et al., 2010). In addition, *HDAC2* expression could be involved in cell proliferation and differentiation in cell type- and developmental stage-specific patterns of expression in the developing cerebellum (Yoo et al., 2013).

*MARCKS* gene is expressed in the brain and spinal cord during embryological development. Stumpo et al. (1995) demonstrated a vital role for *MARCKS* in the normal processes of neurulation, hemisphere fusion, forebrain commissure formation, and formation of cortical and retinal laminations through gene knock-out studies (Stumpo et al., 1995).

Moreover, biallelic partial loss-of-function mutations of *CDC40* gene, which is included in 6q21q22.1 region and encodes a core spliceosomal component, were proven to interfere with RNA splicing and neuronal survival. This was recently associated with pontocerebellar hypoplasia and partial agenesis of the CC with microcephaly in humans and mice. In addition, knock-out of the *CDC40* gene is lethal *in utero* in animal models (Chai et al., 2021).

Haploinsufficiency of multiple genes can be speculated to be involved in brain development and, as such, in the neurological anomalies documented in patients with 6q21q22.1 deletion. Nonetheless, further research and the development of tools and databases focusing on this specific rearrangement are needed to advance our understanding of this genetic mechanism.

## Conclusion

Interstitial deletions of the long arm of chromosome 6 are associated with an extremely variable phenotype. Disease expression also depends on, but is not limited to, the size and the location of the rearrangement. The review of current literature along with this new report can provide further insights on 6q21q22.1 chromosomal deletions and can help to restrict the Smallest Region of Overlap (SRO) associated with peculiar phenotypes.

Even though there is little evidence yet, we speculate that interstitial 6q deletion might be a risk factor for CBVH and/or cerebellar anomalies. These features should be considered by the clinitian who suspects the involvement of 6q21q22.1 rearrangements as the cause of their patient’s phenotype. We recommend neurological follow-up for patients with 6q interstitial deletions, as neurological symptoms may become evident over time. Further investigation on this aspect is needed in a larger group of genetically confirmed 6q interstitial deletion patients, as cerebellar hypoplasia can run asymptomatically.

## Data Availability

The datasets for this article are not publicly available due to concerns regarding participant/patient anonymity. Requests to access the datasets should be directed to the corresponding author.
